# Implications of maraviroc and/or rapamycin in a mouse model of fragility

**DOI:** 10.18632/aging.103167

**Published:** 2020-04-30

**Authors:** Laura Pérez-Martínez, Lourdes Romero, Sandra Muñoz-Galván, Eva M. Verdugo-Sivianes, Susana Rubio-Mediavilla, José A. Oteo, Amancio Carnero, José-Ramón Blanco

**Affiliations:** 1 Centro de Investigación Biomédica de La Rioja (CIBIR), Logroño, España; 2 Instituto de Biomedicina de Sevilla, IBIS, Hospital Universitario Virgen del Rocío, Universidad de Sevilla, Consejo Superior de Investigaciones Científicas, Sevilla, España; 3 CIBERONC, Instituto de Salud Carlos III, Madrid, España; 4 Servicio de Anatomía Patológica, Hospital San Pedro, Logroño, España; 5 Servicio de Enfermedades Infecciosas, Hospital San Pedro, Logroño, España

**Keywords:** CCR5 antagonist, frailty, myostatin, rapamycin

## Abstract

Background: As age increases, the risk of developing fragility also increases. Improving the knowledge of frailty could contribute to maintaining the functional ability of elderly people. Interleukin (IL)-10 homozygous knockout mice (IL-10^tm/tm^ [IL10KO]) constitute an excellent tool for the study of frailty. Because patients with frailty demonstrate an overexpression of CCR5, rapamycin (RAPA) and/or maraviroc (MVC), two molecules able to decrease CCR5 expression, were evaluated.

Results: Muscle myostatin was reduced in all the therapeutic groups but the MVC group (p <0.001 for RAPA and MVC-RAPA) and in serum samples (p <0.01 for all the groups). Serum CK levels were also significantly lower in MVC and RAPA groups (p <0.01 in both cases). Lower AST levels were observed in all the therapeutic groups (p <0.05 for all of them). The apoptotic effector caspase-3 was significantly lower in MVC and RAPA groups (p<0.05 in both cases). Combined treatment with MVC-RAPA showed a synergistic increase in p-AKT, p-mTOR and SIRT1 levels.

Conclusions: MVC and RAPA show a protective role in some factors involved in frailty. More studies are needed to prove their clinical applications.

Material and methods: Eighty male homozygous IL10KOs were randomly assigned to one of 4 groups (n= 20): i) IL10KO group (IL10KO); ii) IL10KO receiving MVC in drinking water (MVC group), iii) IL10KO receiving RAPA in drinking water (RAPA group), and finally, iv) MVC-RAPA group that received MVC and RAPA in drinking water. Blood and muscle samples were analysed. Survival analysis, frailty index calculation, and functional assessment were also performed.

## INTRODUCTION

Longevity is one of the greatest achievements of modern societies. By 2050 it is estimated that nearly a quarter of the global population will be over 60 years old [1]. As age increases, the risk of developing fragility also increases, suggesting an association between them [2]. Frailty is a syndrome characterized by a state of increasing vulnerability, decreased physical function, and adverse outcomes (mortality, disability, and hospitalization) [3, 4]. The physical phenotype of frailty, described by Fried et al. [4], shows significant overlap with sarcopenia, a progressive and generalized skeletal muscle disorder that involves the accelerated loss of muscle mass and function [5].

Improving the knowledge of frailty could contribute to maintaining the functional ability of elderly people. However, so far, there are many unanswered questions about this syndrome. Because clinical studies of frailty are limited by ethical, logistical, and biological complications, among others, animal models are contributing greatly to our understanding of the biology of aging and have been used to test new potential interventions to enhance survival [6]. Interleukin (IL)-10 homozygous knockout mice (IL-10^tm/tm^ [IL10KO]) [7] are one of these models and constitute an excellent tool for the study of frailty [8]. This is because IL10KO develop several frailty features such as sarcopenia, muscular weakness, weight loss as well an increase in multiple pro-inflammatory cytokines [8].

Increasing evidence suggests that aging is a regulated process, and its course can be modified by the modulation of signal transduction pathways [9]. To date, no specific drugs have been approved for the treatment of frailty but this is a major area of research. Rapamycin (RAPA), a macrolide antibiotic with antiproliferative properties [10], is a specific inhibitor of the mechanistic target of rapamycin (mTOR) pathway [11]. RAPA, not only extended the life span of mice but also has a variety of aging-related conditions in old mice [11–14]. Indeed, RAPA is able to decrease CCR5 mRNA expression [15, 16], which is overexpressed in patients with frailty [17]. In this sense, CCR5 antagonists could also be a therapeutic option for the treatment of frailty. So far, only one specific CCR5 antagonist is currently approved for clinical use, maraviroc (MVC) [18]. At the moment, it is unknown wheter the combination of RAPA plus MVC could have a synergic effect, but a synergic effect was observed with other non-approved CCR5 antagonists [19, 20]. To our knowledge, no study has evaluated the role of MVC and/or RAPA in persons with frailty.

In the current study, our aim was to evaluate the effects of MVC and/or RAPA in an experimental mouse model of frailty.

## RESULTS

### Survival was similar in all groups

**Figure 1:**
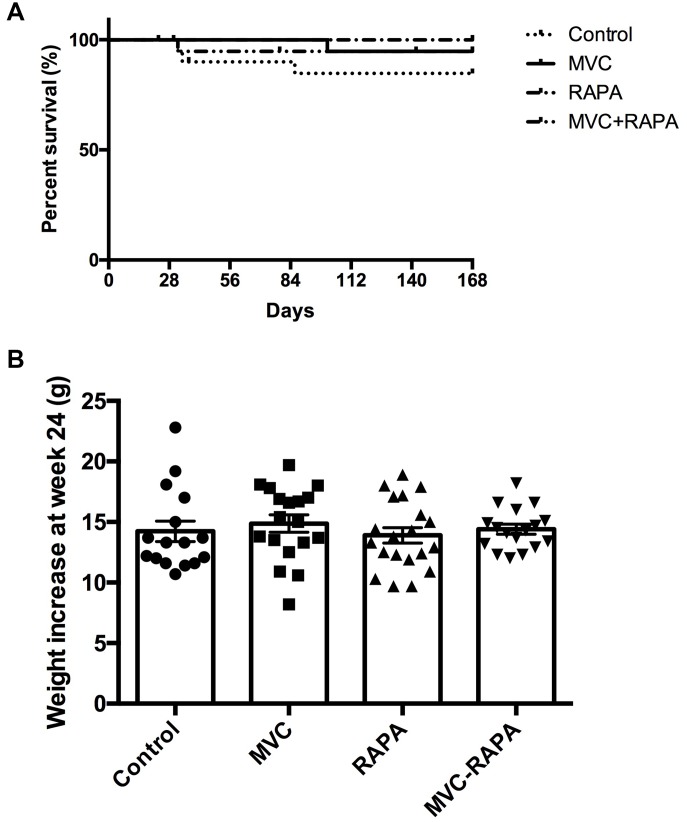
**Survival and body weight.** (**A**) Kaplan-Meier survival plot shows no differences. (**B**) Weight increase did not show statistically significant differences between groups. Each bar represents the mean ± SEM. *p <0.05 with respect to control. MVC, maraviroc. RAPA, rapamycin. MVC+RAPA, maraviroc plus rapamycin.

At the end of the experiment all the groups had a similar survival rates. However, there was a trend towards a greater survival in the RAPA group (p=0.072) (Figure 1).

### None of the therapeutic interventions reduced body weight

The four groups had a similar baseline weight. At the end of the experiment, no significant differences were observed in the body weights (Figure 1).

### All the therapeutic interventions reduced the transaminases

**Figure 2:**
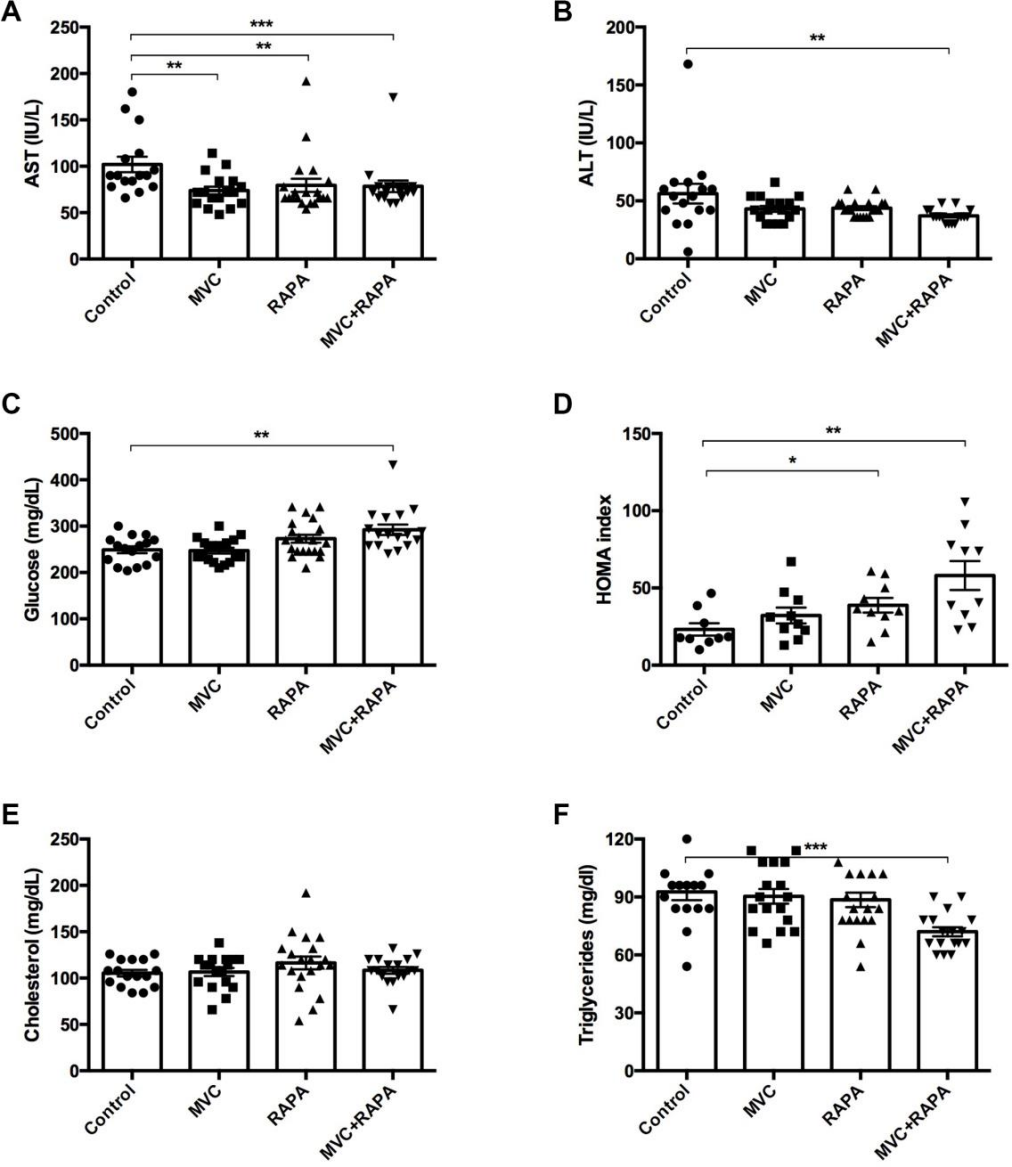
**Biochemical and metabolic parameters: AST, ALT, glucose, HOMA index, cholesterol, triglycerides.** (**A**) A significant decrease in AST levels was recorded in all the therapeutic groups. (**B**) There was a decrease in ALT levels in all the therapeutic groups but only statistically significant in the MVC+RAPA group. (**C**) Glucose levels were discreetly superior in all the therapeutic groups, but only significant in the MVC+RAPA group. (**D**) Compared to control group, HOMA index was significantly higher in RAPA and MVC+RAPA group. (**E**) No differences were observed in the groups after analyzing the cholesterol levels. (**F**). Compared to control group, significantly differences were only observed after comparing it to the MVC+RAPA group. Each bar represents the mean ± SEM. *p <0.05, **p <0.01 and ***p <0.001 with respect to control. ALT, alanine aminotransferase. AST, aspartate aminotransferase. MVC, maraviroc. RAPA, rapamycin. MVC+RAPA, maraviroc plus rapamycin.

Extra-hepatic sites of aminotransferases include skeletal muscle [31]. For this reason, myocyte injury could raise both AST and ALT [32]. Significant differences in AST levels were observed in favor of all the therapeutic groups: MVC (p=0.023), RAPA (p<0.002) and MVC-RAPA (p<0.001). The analysis of ALT also showed a reduction in all the therapeutic groups, mainly in the MVC-RAPA group (p<0.004) and with a clear tendency in the MVC (p=0.085) and RAPA (p=0.076) groups (Figure 2).

### Glucose and insulin parameters were modified in all the groups

Significantly higher serum glucose levels were observed in the MVC-RAPA group (p<0.003). After analyzing the HOMA index, higher levels were observed in the RAPA (p=0.035) and MVC-RAPA (p<0.003) groups (Figure 2). Glucose and insulin tolerance test showed no differences (data not shown).

### The lipid profile of serum TGD improved only in the MVC-RAPA group

No differences were observed after analyzing serum TC levels. Meanwhile, MVC-RAPA groups showed significantly lower levels of serum TGD (p=0.0001) (Figure 2).

### None of the therapeutic interventions improved the weight of the quadriceps or gastrocnemius but CK and myostatin levels were improved

**Figure 3:**
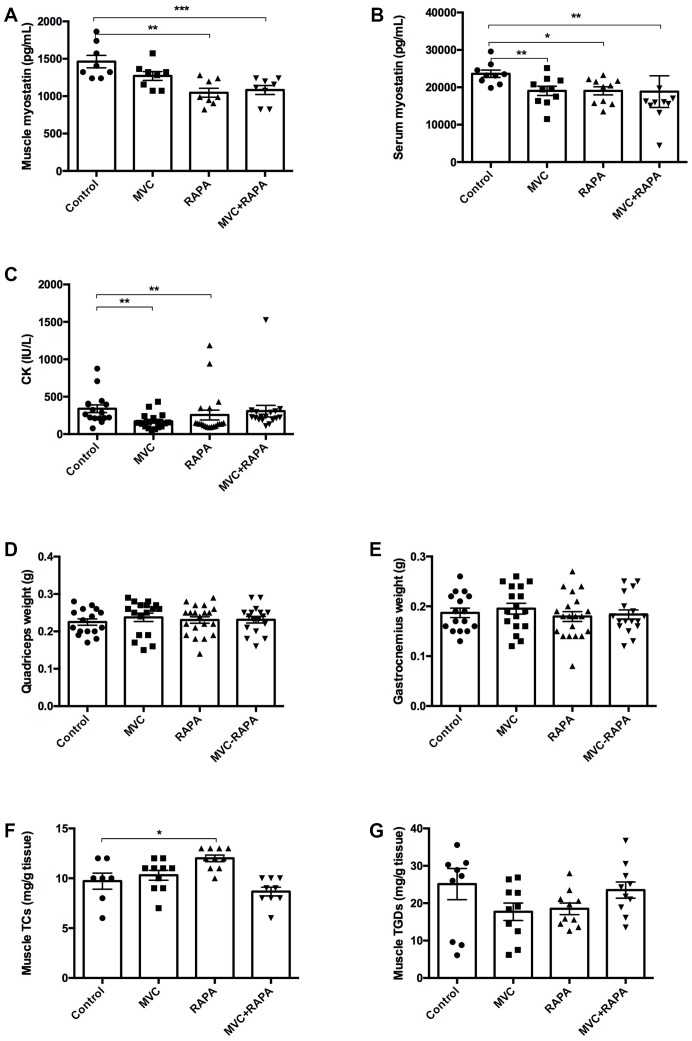
**Biochemical and parameters related to muscle: Muscle and serum myostatin, CK, quadriceps and gastrocnemius weight, muscle cholesterol and triglyceride content.** (**A**) A significant decrease in muscle myostatin levels was recorded in RAPA and MVC+RAPA groups. (**B**) There was a significative decrease in serum myostatin levels in all the therapeutic groups. (**C**) CK was significantly reduced in MVC and RAPA groups. No differences were observed after comparing the different groups for (**D**) quadriceps or (**E**) gastrocnemius weight. (**F**) Muscle cholesterol content was significantly higher in the RAPA groups. (**G**) Although muscle triglyceride content was lower in all the therapeutics groups, no differences were observed. Each bar represents the mean ± SEM. *p <0.05, **p <0.01 and ***p <0.001 with respect to control. CT, cholesterol total. CK, creatinkinase. MVC, maraviroc. MVC+RAPA, maraviroc plus rapamycin. RAPA, rapamycin. TGD, triglyceride.

Regarding myostatin, its inhibition can induce skeletal muscle hypertrophy, while its overexpression causes muscle atrophy [33]. In our study, all the groups but the MVC group showed a statistical reduction in myostatin after analyzing its expression in muscle; RAPA (p=0.001) and MVC-RAPA (p<0.0001). Similar findings were observed after analyzing serum samples; MVC (p<0.01), RAPA (p=0.01), and MVC-RAPA group (p=0.002). The levels of serum CK levels, a sensitive marker of muscle injury, were significantly lower in the MVC (p<0.002) and RAPA (p<0.01) groups. Nonetheless, the four groups had similar weights at the end of the experiment, and no significant differences were observed. Finally, contrary to expectations, muscle TC levels were significantly higher in the RAPA group (p=0.012), but no differences were observed after analyzing muscle TGD levels (Figure 3).

### Skeletal muscle signaling showed differences only after analyzing caspase-3

**Figure 4:**
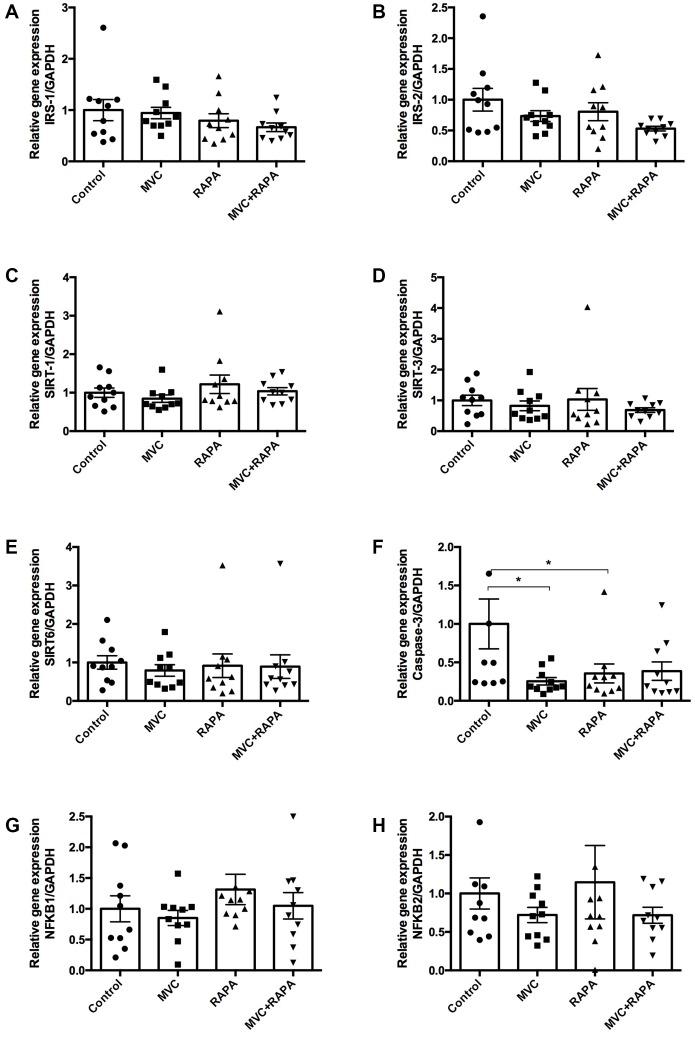
**Muscle expression of insulin receptor substrate (1, 2), sirtuin (1-3), caspase-3, and nuclear factor KB at the RNA level.** No differences were observed after analyzing (**A**) IRS-1, (**B**) IRS-2, (**C**) SIRT-1, (**D**) SIRT-3, or (**E**) SIRT-6. (**F**) Although caspase-3 was lower in all the therapeutics groups it was only statistically significant in MVC and RAPA groups. No differences were observed after analyzing (**G**) NFKB-1 and (**H**) NFKB-2. Each bar represents the mean ± SEM. *p <0.05 with respect to control. IRS, insulin receptor substrate. MVC, maraviroc. MVC+RAPA, maraviroc plus rapamycin. NFKB, nuclear factor KB. RAPA, rapamycin. SIRT, sirtuin.

IRS (1 and 2) showed no differences, although MVC-RAPA showed a tendency towards a lower level of IRS-2 (p=0.063). In the same way, SIRT (1, 3 and 6) showed no differences. Meanwhile, caspase-3, an inducer of apoptosis, was significantly lower in the MVC (p=0.011) and RAPA groups (p<0.05), showing also a clear tendency in the MVC-RAPA group (p=0.063). Finally, NFKB (1 and 2) showed no differences (Figure 4).

### Effects on cytokines or chemokines

**Figure 5:**
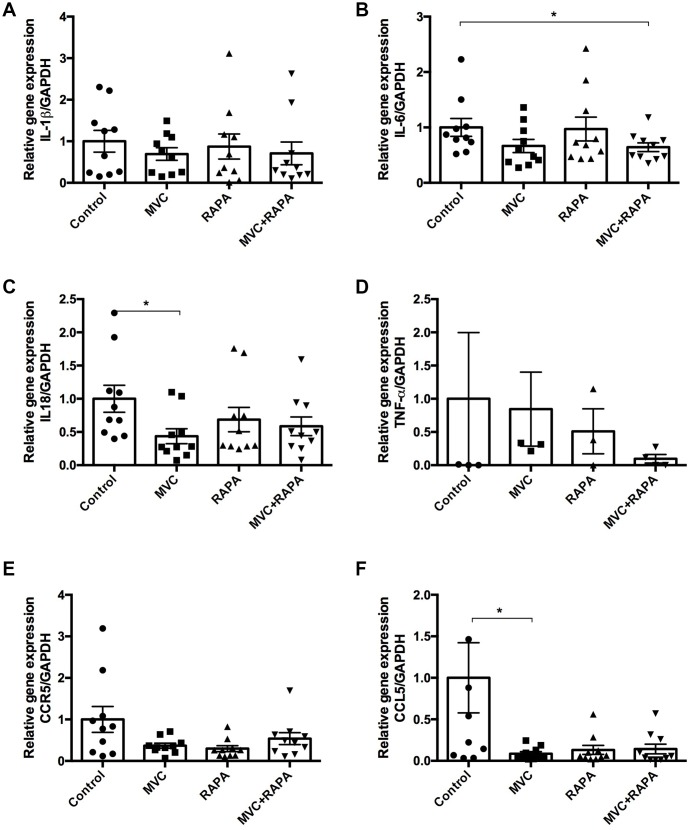
**Muscle expression of IL-1β, IL6, IL-18, TNF-α, CCR5 and CCL5 at the RNA level.** (**A**) No differences were observed after analyzing IL-1β. (**B**) IL-6 expression was significantly lower in the MVC+RAPA group. (**C**) Although IL-18 expression was lower in all the therapeutic groups, it was only statistically significant in the MVC group. (**D**) Although TNF-α and (**E**) CCR5 expression were lower in all the therapeutic groups, none of them was statistically significant. (**F**) CCL5 expression was significantly lower in the MVC group and showed a clear tendency to statistical significance in the RAPA and MVC-RAPA group. Each bar represents the mean ± SEM. *p <0.05 with respect to control. IL, interleukin. MVC, maraviroc. MVC+RAPA, maraviroc plus rapamycin. RAPA, rapamycin. TNF-α, tumor necrosis factor-alpha.

In this study, no differences were observed after analyzing muscle IL-1β. However, IL-6 levels were significantly lower in the MVC-RAPA group (p=0.028) and showed a tendency in the MVC group (p=0.089). Likewise, in muscle samples, IL-18 levels, another pro-inflammatory cytokine, were significantly lower in the MVC groups (p=0.014). No differences were observed after analyzing TNF-α. In the same way, no differences were observed after analyzing serum IL-1β, IL-6, or TNF-α (data not shown). In reference to chemokines, no differences in muscle CCR5 expression were observed after analyzing the different groups although there was a clear tendency in the RAPA group (p=0.054). Similarly, muscle CCL5 expression was significantly lower in the MVC group (p=0.035) and showed a clear tendency in the RAPA and MVC-RAPA groups (p=0.063 and p=0.052, respectively) (Figure 5).

### WB analysis showed

**Figure 6:**
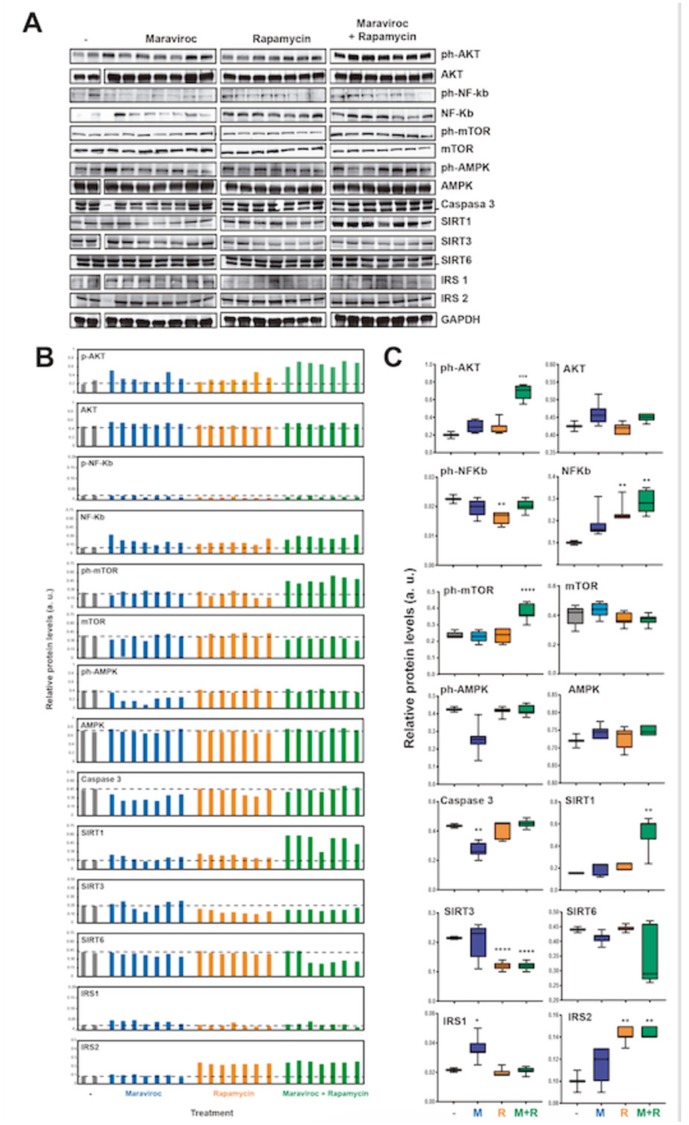
Analyses of the molecular pathways involved in the mechanism of action of RAPA and MVC (**A**) Western blot analysis of the protein levels of AKT (phosphorylated and total), NF-kb (phosphorylated and total), mTOR, AMPK (phosphorylated and total), Caspase 3, SIRT1, SIRT3, SIRT6, IRS1, IRS2 and GAPDH as endogenous control, in mice treated or not with maraviroc (MVC group), Rapamycin (RAPA group) or combination of both (MVC-RAPA group). (**B**) Relative quantification of the protein levels in (**A**) relative to GAPDH control. (**C**) Box plots showing the average protein levels from (**B**). Data were analyzed using Student t test´s. *, P < 0.05; **, P < 0.01; ***, P < 0.001; ****, P < 0.0001.

That mice treated with MVC showed a significant decrease in caspase-3 (p <0.01) and IRS1 (p <0.05). In addition, the RAPA group showed a decrease in p-NFKB (p <0.01) and SIRT3 (p <0.0001) but an increase in total NFKB (p <0.01) and IRS2 (p <0.01). The MVC-RAPA group showed a significant increase in the p-AKT (p <0.001), total NFKB (p <0.01), p-mTOR (p <0.0001), SIRT1 (p <0.01) and IRS2 (p< 0.01), but a decrease in SIRT3 (Figure 6). It is worth noting that the decrease in caspase-3 protein upon MVC treatment was also observed at the mRNA level (Figure 4), while other effects may be different at the protein and mRNA levels due to differences in translation efficiency and/or protein stability. Interestingly, MVC-RAPA group showed a synergistic effect on p-AKT and SIRT1 protein levels, which are increased only by their combined treatment.

### Histological assessment of skeletal muscle

H&E and Masson’s trichrome staining showed no differences in any of the four groups (data not shown).

### No differences were observed after analyzing frailty parameters

**Figure 7:**
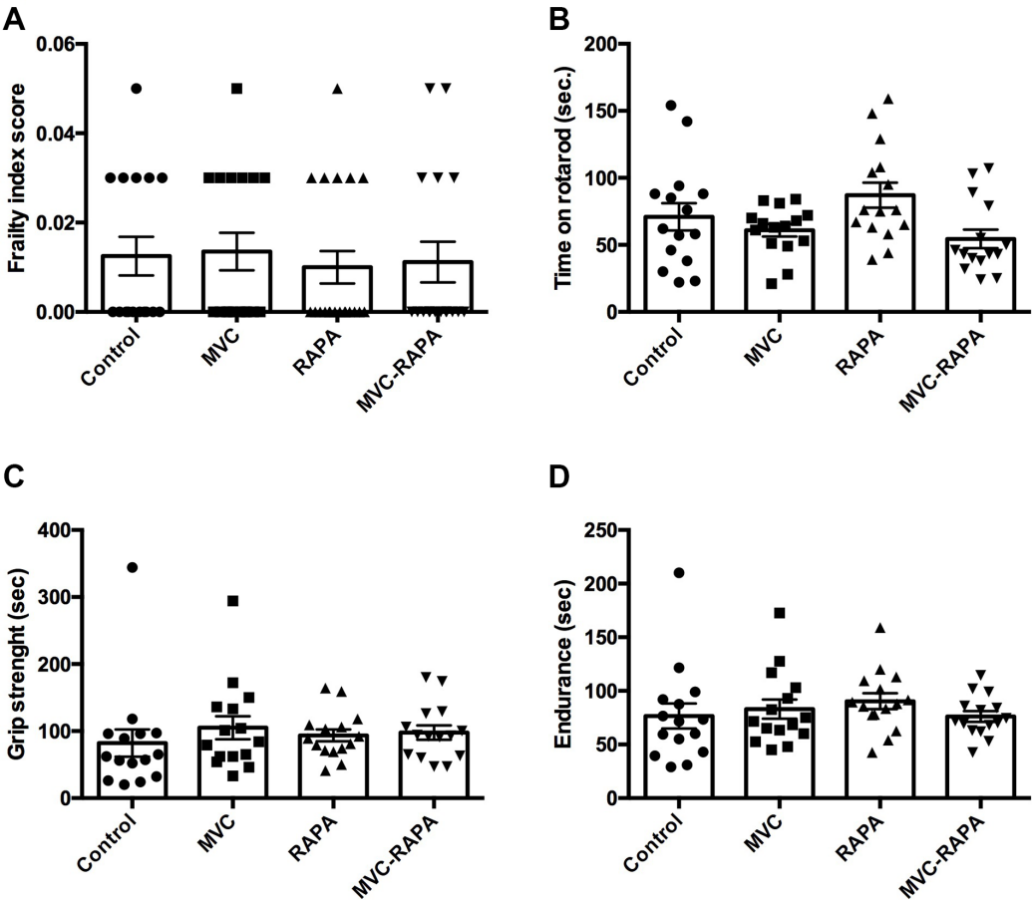
**Frailty parameters: frailty index score, time on rotarod, grip strength, and endurance.** No differences were observed after analyzing any of the frailty parameter for (**A**) frailty index score, (**B**) time on rotarod, (**C**) grip strength, and (**D**) endurance. Each bar represents the mean ± SEM. MVC, maraviroc. MVC+RAPA, maraviroc plus rapamycin. RAPA, rapamycin.

At the end of the experiment, no differences were observed after analyzing the frailty index score, time on rotarod, grip strength or endurance. However there was a favorable trend in grip strength in the RAPA (p=0.095) and MVC-RAPA (p=0.091) groups. In addition, endurance also showed a tendency towards improvement in the RAPA group (p=0.097) (Figure 7).

## DISCUSSION

To properly manage frailty it is necessary to improve the knowledge of this syndrome. To reduce frailty, exercise programs and nutritional supplementation have been proposed as strategies to prevent and treat frailty and sarcopenia [34]. Now, in this animal model, we show interesting data about the possibility of adding a therapeutic strategy such as MVC.

First, we have shown the beneficial effects of MVC and RAPA on myostatin, a negative regulator of muscle mass [35] that has been implicated in wasting conditions such as sarcopenia and cachexia [36]. In addition to the effects of myostatin on muscle mass, muscle myostatin deficiency has beneficial effects on metabolism, adiposity and insulin sensitivity [37]. Although previous studies have shown that muscle myostatin was not affected by RAPA administration [38], we have observed a decrease in its levels. To our knowledge, this effect has not been described previously for MVC.

Another issue is the controversial relationship between serum levels of myostatin and frailty. Regarding older adults, some authors [39, 40] observed a direct correlation between serum myostatin levels and skeletal muscle mass, while others [41] found that muscle mass was inversely correlated with serum myostatin levels. The differences in the results are mainly related to the assessment tool used to characterize frailty. In our project, we observed a decrease in the serum and muscular levels of myostatin in all therapeutic groups. As far as we know, we have not found studies that correlate serum and muscle levels of myostatin; therefore this study could also provide more clarity to the existing controversy.

Second, levels of serum CK, a sensitive marker of muscle injury [42], were significantly lower in the MVC and RAPA groups. In line with this observation and as far as we know, in the literature, there is a report

of and elderly HIV-infected patient with disabling inflammatory myopathy who had a reduction of his myositis after starting MVC [43], which would suggest its beneficial effect at the muscular level. No references have been found about the potential beneficial effect of RAPA on serum CK levels.

Third, AST levels, and in some way ALT levels, were lower in all the therapeutic groups. No references have been found about the potential beneficial effect of RAPA on AST levels. Although AST and ALT are important hepatotoxic biomarkers, they are also a biochemical marker of muscular damage [42]. Therefore, in chronic muscle injury, AST and ALT are both increased. In a study performed in the general population, sarcopenia was a risk factor for elevated aminotransferase in men independent of body mass index, dietary habits, and physical activity [44].

Fourth, caspases are crucial mediators of apoptosis. In this study, nearly all the therapeutic groups, in particular the MVC group, showed lower levels of caspase-3. This decrease in caspase-3 expression upon MVC treatment was also observed at the protein level. Previous studies showed an upregulation of caspase activity and apoptosis in old rats [45] and elderly persons [46]. Indeed, because apoptotic death is observed in multiple myopathies, it has been suggested that its inhibition could be a potential therapeutic strategy in the treatment of these disorders [47]. It will be necessary to evaluate the clinical potential of our observations.

All these observations support the idea that MVC and RAPA, both of which are CCR5 antagonists, could protect against muscle damage. In this sense, in an animal model of progressive muscle weakness, such as Duchenne muscular dystrophy, Liang et al. [48] observed that cenicriviroc (CVC), a dual chemokine receptor (CCR2/CCR5) antagonist, could slow disease progression. In their experiment they observed an important reduction in total infiltrating macrophages but not changes in muscle fiber size or fibrosis. In addition, the authors also observed an increase in the maximal isometric force production with CVC therapy [48].

Previous studies have shown the ability of MVC to ameliorate the increased adipose tissue macrophage recruitment induced by a high-fat-diet mouse model of obesity [49]. Something similar was observed employing a murine model of genetic dyslipidaemia. In this case, MVC was able to inhibited atherosclerotic progression, among others, by reducing macrophage infiltration [50].

Against what we would have expected, in this animal model, we did not observe changes in the main cytokine levels. Therefore, it is possible that the potential protective effect of MVC and RAPA in this model of frailty does not depend on a pro-inflammatory or anti-inflammatory route. This is an aspect of interest because, at least in humans, the CCR5 RNA expression of skeletal muscle is very low (approximately 4 and 30 times lower than corresponding levels in the liver and peripheral blood lymphocytes, respectively) [51]. Indeed, despite the fact that both MVC and RAPA have anti-CCR5 effects, each of them also has a different impact on most of the tests analyzed, with some notable exceptions (i.e. AST, myostatin).

Finally, it is known that RAPA administration decreases CCR5 mRNA expression both *in vitro* [15] and *in vivo* (macaques) [16]. In addition, RAPA induced a synergistic enhancement of the CCR5 antagonists effects of vicroviroc [19] and aplaviroc [20] against HIV. To our knowledge, no studies have been performed on the synergistic effect of RAPA plus MVC. In this animal model, we did not observe a synergistic, additive or antagonistic effect on the level of CCR5 expression in the MVC-RAPA group. However, a synergistic increase in p-AKT, p-mTOR and SIRT1 protein levels upon MVC-RAPA treatment was observed, suggesting that they could have a protective effect.

This study could have some limitations. First, life extension by RAPA is more prominent at higher doses [14] than the dose we have employed. However, our objective was not to improve survival. Indeed, there is a concern about potential side effects (i.e., glucose intolerance or insulin resistance) [52, 53] that may limit MVC-RAPA use as an anti-aging drug. Because it is known that deficiency of CCR5 impairs systemic glucose tolerance [54], the double impact on CCR5 (MVC plus RAPA) may be the reason for the highest increases in the HOMA index.

In summary, our data could support that MVC and RAPA have a protective role in some factors involved in the development of frailty. These data could justify a randomized, controlled trial to determine their beneficial effects on patients with frailty.

## MATERIALS AND METHODS

### Animals and animal models

A total of 80 male homozygous IL-10 deficient mice (B6.129P2-IL10^tm1Cgn^/J) were purchased from Jackson Laboratory (Bar Harbor, ME, USA). All animals were housed in pathogen-free barrier conditions and had free access to food and drinking water during the study. When the animals were approximately 6 weeks old, they were randomly assigned (n = 20) to one of 4 groups and fed for 24 weeks: i) the IL-10KO group (IL-10KO) received a standard rodent diet and tap water; ii) the preventive MVC group received the same diet as the IL-10KO group and received MVC (Pfizer, New York, NY) in their drinking water (300 mg/L) [21–23]; iii) the preventive RAPA group [24] received the same diet as the IL-10KO group and received RAPA in their drinking water (1.5 mg/kg/day) [25]; and iv) the preventive MVC plus RAPA group (MVC-RAPA) received the same diet as the IL-10KO group and received MVC plus RAPA in their drinking water at the same concentration as the MVC or RAPA group.

The mice were observed daily, and all the observations were recorded. In addition, the animals were weighed once a week. All the animals were sacrificed at week 24. At that time, blood samples were collected under anaesthesia after a 4-hour fasting period.

### Blood sampling and analysis

Plasma levels of aspartate aminotransferase (AST), alanine aminotransferase (ALT), glucose, triglycerides (TGD), cholesterol (TC) and creatine kinase (CK) were measured using an automatic biochemical analyzer (Cobas C711, Roche, Madrid, Spain). Insulin resistance and insulin sensitivity were determined by using the homeostasis model assessment of insulin resistance (HOMA-IR) [26]. Glucose tolerance test was measured 14 days before sacrificing the animals [27]. Serum myostatin levels were measured by using an ELISA kit (R&D Systems).

### Muscle preparation

Skeletal muscle tissues (quadriceps and gastrocnemius) were excised, weighed and frozen in liquid nitrogen and stored at -80 °C until processing.

To quantify muscle TGD and TC content, 150 mg of muscle tissue was homogenized in 1.5 mL of buffer (150 mM NaCl, 0.1% Triton X-100 and 10 mM Tris pH 8) at 50ºC using an Ultraturrax (IKA-Weke, Staufen, Germany) homogenizer. After the centrifugation of the homogenate at 12,000 g for 10 minutes, an autoanalyser was used to measure the TGD and TC levels in the obtained supernatant. Muscle myostatin levels were measured by using an ELISA kit (R&D Systems).

### Gene expression quantification

Total RNA was extracted and purified from muscle samples using an RNA RNeasy Mini Kit (Qiagen, Valencia, CA), and was treated with DNase I (Qiagen) following the manufacturer’s instructions. cDNA was synthesized by reverse transcription of 1 μg of total RNA using the SuperScript III First-Strand Synthesis kit (Invitrogen) in a total volume of 30 μl according to the manufacturer’s instructions, followed by amplification using SybrGreen (Takara Bio Inc., Shiga, Japan) PCR.rt using specific primers (Supplementary Table 1).

Amplification and detection of specific products were performed using the ABI PRISM 7300 (Applied Biosystems, Foster City, CA, USA). All reactions were run in duplicate for each studied sample. The relative expression of the biomarkers analyzed was calculated according to the manufacturers’ instructions. All the results were divided by their corresponding housekeeping gene value.

### Sandwich enzyme linked immunosorbent assay (ELISA)

TNF-α, IL-1β, IL-6 and myostatin levels were quantified in serum samples (Supplementary Table 2). The serum was separated from the cells after clot formation by centrifugation. For each assay performed, a minimum of 2 wells must be used as blanks; therefore, primary antibodies were not applied to those wells. The ELISA plate (Nunc Maxisorp) was coated with 100 μl/well of capture antibody in 1X Coating Buffer. The plate was sealed and incubated overnight at 4°C. All wells were aspirated and washed 5 times with 300 μl/well of Wash Buffer.

Afterwards, the wells were blocked with 200 μl/well of 1X ELISA Diluent and incubated at room temperature for 1 hour. To activate the latent antibody to its immunoreactive form, the samples (but not the standards) were acidified and then neutralized. The mouse serum was diluted 1:8 in PBS. Per 100 μl of sample, 20 μl of 1N HCl was added and incubated for 10 minutes at room temperature and then neutralized with 20 μl of 1N NaOH. Using 1X ELISA Diluent, 2-fold serial dilutions of the top standards were performed to make a standard curve. A volume of 100 μl/well of the standard dilutions was added to the appropriate wells. In the same way, 100 μl/well of the acid-activated samples was added to the appropriate wells. The plate was sealed and incubated at room temperature for 2 hours. After this time, the wells were aspirated and washed 4 times with 300 μl/well of Wash Buffer. Then, 100 μl of detection antibody diluted in 1X ELISA Diluent was added to each well and incubated at room temperature for 1 hour. All wells were aspirated and washed 5 times. A volume of 100 μl/well of Avidin-HRP diluted in 1X ELISA Diluent was added and the plate was incubated at room temperature for 30 minutes. The wells were aspirated and washed 7 times. A volume of 100 μl of 1X TMB Solution was added to each well and the plate was incubated at room temperature for 15 minutes. Afterward, 50 μl of Stop Solution was added to each well. The plate was read at 450 nm and 570 nm.

### Western blot analysis

Muscle samples were lysed using a homogenizer and treated with RIPA lysis buffer (Sigma Aldrich, St. Louis, MO). The cell lysate was centrifuged at 10,000 g for 10 minutes at 4ºC. The concentration of total protein in each sample was determined by the Bradford method.

### 5'-Adenosine monophosphate-activated protein kinase (AMPK), phosphorylated

AMPK (pAMPK), protein kinase-B (Akt), phosphorylated AKT, nuclear factor-kB (NFKB), phosphorylated NFKB (pNFKB), mTOR, phosphorylated mTOR, sirtuin (SIRT) (1, 3, and 6), insulin receptor substrates (IRS) (1 and 2), and caspase-3 were evaluated by Western blotting (WB). GAPDH was used as a control (Supplementary Table 3).

Samples (30 μg per well) were subjected to polyacrylamide gel electrophoresis (SDS–PAGE). Subsequently, the proteins were transferred to a nitrocellulose membrane that was incubated with a specific antibody against the proteins of interest. Proteins were detected by colorimetry using a secondary antibody bound to the peroxidase enzyme (Anti-rabbit IgG, Cell Signaling, Danvers, MA) and incubation with the corresponding substrate. After high-resolution scanning, the concentration of each protein was evaluated by densitometry using ImageJ software. The density values for each of the test samples were normalized based on the values of GAPDH, which was used as a loading control.

### Muscle fiber staining

Following fixation, skeletal muscle tissues (quadriceps and gastrocnemius) were dehydrated and paraffin embedded. Tissue sections (3 μm-thick) were rehydrated and stained according to standard protocols with hematoxylin and eosin (H&E) and Masson’s trichrome staining, which stains muscle fibers red, nuclei black, and collagen blue. Muscle fiber size was measured using ImageJ software.

### Survival study

Animals were inspected daily for health issues, and deaths were recorded for each animal. Mice were euthanized for humane reasons if so severely moribund that they were considered by an experienced technician, unlikely to survive for more than an additional 48 hours. Moribund animals were euthanized by CO_2_ asphyxiation and recorded. Every animal found dead or euthanized was necropsied. Criteria for euthanasia were considered by an experienced technician, according to the AAALAC guidelines. For the longevity study, only cases where the condition of the animal was considered incompatible with continued survival are represented as deaths in the curves. Animals removed at sacrifice or euthanized due to reasons not related to incompatible survival were considered censored deaths.

### Frailty index assessment

A frailty index (FI) score was calculated for each mouse using the 31-item FI [28]. The sum of the scores for 31 items was divided by 31 to give the FI. The validity of this scale was correlated with human FI and had a good agreement [28]. The temperature and weight scores were calculated based on the number of standard deviations of the tested mouse compared with the reference values from sex-matched adult animals.

### Functional assessment

The inverted-cling grip test was used for evaluating the grip strength of limbs and the endurance of muscles in the mice [29]. This procedure was performed as proposed by Liu et al. [30]. The walking speed was evaluated using the rotarod test (RotaRod R/S; LSi Letica, Cornella, Spain). The rotarod test is widely used for determining overall motor function in mice [29]. This procedure was performed as proposed by Liu et al. [30]. Finally, both the grip test and the rotarod test were used to assess overall endurance [29]. The equation employed was [(Endurance score (seconds) = (Time of grip test + Time of rotarod test)/2)].

### Statistics

Data are presented in the figures as the mean ± SEM (standard error of the mean). Body weight data were analyzed with analysis of variance followed by the Dunnet post-hoc test. For all other data, the Kruskal–Wallis test was used followed by the Mann–Whitney U-test. Correlation between variables was determined using the Spearman rank-sum test. Survival analysis was performed using Kaplan–Meier survival analysis. All data were analyzed with GraphPad Prism 6 software and were considered statistically significant when p<0.05.

### Ethics statement

All procedures were carried out in accordance with the European Communities Council Directive (86/609/CEE) on animal experiments and with approval from the ethical committee on animal welfare of our institution (Comité Etico de Experimentación Animal del Centro de Investigación Biomédica de La Rioja, CEEA-CIBIR).

## Supplementary Material

Supplementary Tables
