# Supplementary material for: Implications of maraviroc and/or rapamycin in a mouse model of fragility
Source: Aging (Albany NY). 2020 Apr 30;12(9):8565–82. doi: 10.18632/aging.103167 (PMC7244075; doi:10.18632/aging.103167)
Supplement: Supplementary Tables [file aging-12-103167-s001..pdf]

SUPPLEMENTARY TABLES

Supplementary Table 1. List of primers used by SybrGreen for real-time PCR.

Name	Gene symbol	Primers Sequence
**Glyceraldehide 3-phoshate dehydrogenase**	**GAPDH**	Sense: 5′-CATGTTCCA GTATGACTCCACTC-3′<br>Antisense: 5′-GGCCTCA CCCCATTTGATGT-3′
**Interleukin-6**	**IL-6**	Sense: 5′-ATGGATGCTA CCAAACTGGAT-3′<br>Antisense: 5′-TGAAGGACTCTGGCTTTGTCT-3′
**Interleukin-1 beta**	**IL-1β**	Sense: 5′-CTGAACTCAACTGTGAAATGCCA-3′<br>Antisense: 5′-AAAGGTTTGGAAGCA GCCCT-3′
**Tumor necrosis factor- α**	**TNF-α**	Sense 5′- ACGGCATGGA TCTCAAAGAC-3′<br>Antisense 5′- AGATAGCAAATCGGCT GACG-3′
**Interleukin-18**	**IL-18**	Sense: 5′- GACTCTTGCGTCAACTTCAAGG -3′<br>Antisense: 5′- CAGGCTGTCTTTTGTCAACGA -3′
**Chemokine receptor type 5**	**CCR5**	Sense: 5′-CGAAAACACATGGTCAAACG-3′<br>Antisense: 5′-TTCCTACTCCCAAGCTGCAT-3′
**Chemokine ligand 5 (RANTES)**	**CCL5**	Sense: 5′-ATATGGCTCGGACACCACTC-3′<br>Antisense: 5′-GTGACAAACACGACTGCAAGA -3′
**Mammalian target of Rapamycin**	**mTOR**	Sense: 5′- CTGGGACTCAAATCTCTCCAGTTC-3′<br>Antisense: 5′- GAACAATAGGGTGAATGATCCGGG-3′
**Caspase-3**	**Caspase-3**	Sense: 5′-ATGGGA GCAA GTCA GTGGAC-3′<br>Antisense: 5′-CGTACCA GA GCGA GATGACA-3′
**Insuline receptor substrate-1**	**IRS-1**	Sense: 5′- CCAGCCTGGCTATTTA GCTG-3′<br>Antisense: 5′-CCCAACTCAACTCCACCACT-3′
**Insuline receptor substrate-2**	**IRS-2**	Sense: 5′-GTAGTTCA GGTCGCCTCTGC-3′<br>Antisense: 5′-CAGCTATTGGGACCACCACT-3′
**NAD-dependent protein deacetylase sirtuin-1**	**SIRT-1**	Sense: 5′- AGTTCCAGCCGTCTCTGTGT-3′<br>Antisense: 5′- CTCCACGAACA GCTTCACAA-3′
**NAD-dependent protein deacetylase sirtuin-3**	**SIRT-3**	Sense: 5′ GCTGCTTCTGCGGCTCTATAC-3′<br>Antisense: 5′-GAAGGACCTTCGACA GACCGT-3′
**NAD-dependent protein deacetylase sirtuin-6**	**SIRT-6**	Sense: 5′- CTGAGAGA CACCATTCTGGACT-3′<br>Antisense: 5′- GGTTGCA GGTTGA CAATGACC-3′
**Nuclear factor NF-kappa-B p105 subunit**	**NFkB1**	Sense: 5′-GAAATTCCTGATCCA GA CAAAAAC-<br>3′Antisense: 5′-ATCACTTCAATGGCCTCTGTGTA G-3′
**Nuclear factor NF-kappa-B p100 subunit**	**NFkB2**	Sense: 5′-CTGGTGGA CA CATACA GGAA GA C-<br>3′Antisense: 5′-ATA GGCACTGTCTTCTTTCA CCTC-3′

Supplementary Table 2. Details of the ELISA assay methods.

Biomarker	Procedure	Sensitivity Minimum Detectable Dose (MDD)	Commercial Producer
IL-1β	Quantikine® ELISA	0.46-4.80 pg/ml (The mean was 2.31 pg/ml)	R&D Systems
IL-6	Quantikine® ELISA	1.3-1.8 pg/ml (The mean was 1.6 pg/ml)	R&D Systems
TNF-α	Quantikine® ELISA	0.36-7.21 pg/ml (The mean was 1.88 pg/ml)	R&D Systems
Myostatin	Quantikine® ELISA	0.922-5.32 pg/ml (The mean was 2.25 pg/ml)	R&D Systems

Abbreviations: 1 IL-1β = interleukin 1β; IL6 = interleukin 6; TNF-α = Tumor necrosis factoralpha.

Supplementary Table 3. Kinases detected in this study.

Antibody used	Commercial Producer	Molecular weight of the protein
anti-AMPK antibody	Abcam, Cambridge, MA, USA (Cat. No. 80039)	62 kDa
Anti-AMPK (phospho Thr 172) antibody	Cell Signaling, Danvers, MA (Cat. No. 109458)	60 kDa
Anti-AKT antibody	Cell Signaling, Danvers, MA (Cat. No. 9272)	60 kDa
Anti- AKT (phospho S473) antibody	Abcam, Cambridge, MA, USA (Cat No. 81283)	56 kDa
Anti-NF-kb p65 antibody	Abcam, Cambridge, MA, USA (Cat No. 16502)	64 kDa
Anti NF-kb p65 (phospho S529) antibody	Abcam, Cambridge, MA, USA (Cat No. 16502)	60 kDa
mTOR	Cell Signaling, Danvers, MA (Cat. No. 2983T)	289 kDa
Anti-mTOR (phospho S2448) antibody	Abcam, Cambridge, MA, USA (Cat No. 109268)	kDa
Anti-SIRT1 antibody	Abcam, Cambridge, MA, USA (Cat No. 32441)	110 kDa
Anti-SIRT3 antibody	Abcam, Cambridge, MA, USA (Cat No. 217319) 28 kDa	
Anti-SIRT6 antibody	Abcam, Cambridge, MA, USA (Cat No. 191385) 39 kDa	
Anti-IRS1 antibody	Abcam, Cambridge, MA, USA (Cat No. 40777) 170 kDa	
Anti-IRS2 antibody	Abcam, Cambridge, MA, USA (Cat No. 134101) 137 kDa	
Anti-pro Caspase-3 antibody	Abcam, Cambridge, MA, USA (Cat No. 32150)	35 kDa
Anti-GAPDH antibody	Thermo Fisher Scientific, Waltham, MA (Cat. No. AM4300)	37 kDa
